# Vancomycin Associated Acute Kidney Injury: A Longitudinal Study in China

**DOI:** 10.3389/fphar.2021.632107

**Published:** 2021-03-08

**Authors:** Pan Kunming, Chen Can, Chen Zhangzhang, Wu Wei, Xu Qing, Ding Xiaoqiang, Li Xiaoyu, Lv Qianzhou

**Affiliations:** ^1^ Department of Pharmacy, Zhongshan Hospital Fudan University, Shanghai, China; ^2^ Department of Nephrology, Zhongshan Hospital Fudan University, Shanghai, China

**Keywords:** vancomycin, acute kidney injury, risk factors, renal recovery, morality

## Abstract

**Background:** Vancomycin-associated acute kidney injury (VA-AKI) is a recognizable condition with known risk factors. However, the use of vancomycin in clinical practices in China is distinct from other countries. We conducted this longitudinal study to show the characteristics of VA-AKI and how to manage it in clinical practice.

**Patients and Methods:** We included patients admitted to hospital, who received vancomycin therapy between January 1, 2016 and June 2019. VA-AKI was defined as a patient having developed AKI during vancomycin therapy or within 48 h following the withdrawal of vancomycin therapy.

**Results:** A total of 3719 patients from 7058 possible participants were included in the study. 998 patients were excluded because of lacking of serum creatinine measurement. The incidence of VA-AKI was 14.3%. Only 32.3% (963/2990) of recommended patients performed therapeutic drug monitoring of vancomycin. Patients with VA-AKI were more likely to concomitant administration of cephalosporin (OR 1.55, 95% CI 1.08–2.21, *p* = 0.017), carbapenems (OR 1.46, 95% CI 1.11–1.91, *p* = 0.006) and piperacillin-tazobactam (OR 3.12, 95% CI 1.50–6.49, *p* = 0.002). Full renal recovery (OR 0.208, *p* = 0.005) was independent protective factors for mortality. Compared with acute kidney injury stage 1, AKI stage 2 (OR 2.174, *p* = 0.005) and AKI stage 3 (OR 2.210, *p* = 0.005) were independent risk factors for fail to full renal recovery.

**Conclusion:** Lack of a serum creatinine measurement for the diagnosis of AKI and lack of standardization of vancomycin therapeutic drug monitoring should be improved. Patient concomitant with piperacillin-tazobactam are at higher risk. Full renal recovery was associated with a significantly reduced morality.

## Introduction

Currently, vancomycin is the first-line treatment for methicillin-resistant *Staphylococcus aureus* (MRSA) infections. However, it has also been associated with significant acute kidney injury (AKI) (Chen et al., 2011; Liu et al., 2011), which is a common disorder with a high risk of mortality, the development of chronic kidney disease, and substantial medical expense (Yang et al., 2015). There is considerable variation in the incidence of reported vancomycin-associated AKI (VA-AKI), which ranges from 5 to 43% (van Hal et al., 2013). There are numerous potential risk factors for VA-AKI including race, obesity, vancomycin exposure, pre-existing kidney disease, severity of illness, concurrent nephrotoxin exposure, concurrent piperacillin-tazobactam use, etc. (Filippone et al., 2017). However, due to variations in study populations and sample sizes, different studies have identified conflicting risk factors. Several studies have shown that specific races (e.g., African-Americans) have a higher risk for VA-AKI (Bosso et al., 2011; Womble et al., 2019); although, studies specifically investigating Asian populations are lacking. For patients developed VA-AKI, how to reduce mortality and improve renal recovery is still a difficult problem to be explored.

We previously have reported that current literature on VA-AKI mainly came from American hospitals (Pan et al., 2019). However, the clinical use of vancomycin in China is distinct from other countries. For instance, vancomycin and piperacillin-tazobactam are among the most commonly prescribed antibiotics in American hospitals, which are associated with significant increases in the incidence of AKI compared to vancomycin monotherapy or other empirical combinations (Balci et al., 2018; Carreno et al., 2018; Ide et al., 2019; Avedissian et al., 2020; Ciarambino et al., 2020). In contrast, previous studies have shown that, in China, the most common antibiotic combinations with vancomycin are carbapenems (Pan et al., 2017; Pan et al., 2018). Liang et al. found that vancomycin nephrotoxicity was significantly correlated with the trough concentration and reported the first cut-point as 13 mg/L for the Chinese population (Liang et al., 2018). This was in contrast to trough concentrations exceeding 15 mg/L cited in American guidelines (Rybak et al., 2009; Ye et al., 2016). (Yang et al., 2015). found that, in China, a higher proportion of nephrotoxic drug exposure (71.6%) occurred before or while AKI develops as opposed to what has been reported by developed countries (20–50%) (Yang et al., 2015). Therefore, we designed this cohort study to include large sample patients, who are widely distributed and included a comprehensive number of risk factors. We believe that data from China, the most populous country in Asia, and the world’s largest developing nation, will provide valuable information for assessing the burden of VA-AKI in this population, as well as describe its clinical characteristics, show how to recognize and manage VA-AKI in clinical practice.

## Methods

### Study Design and Patient Population

**FIGURE 1:**
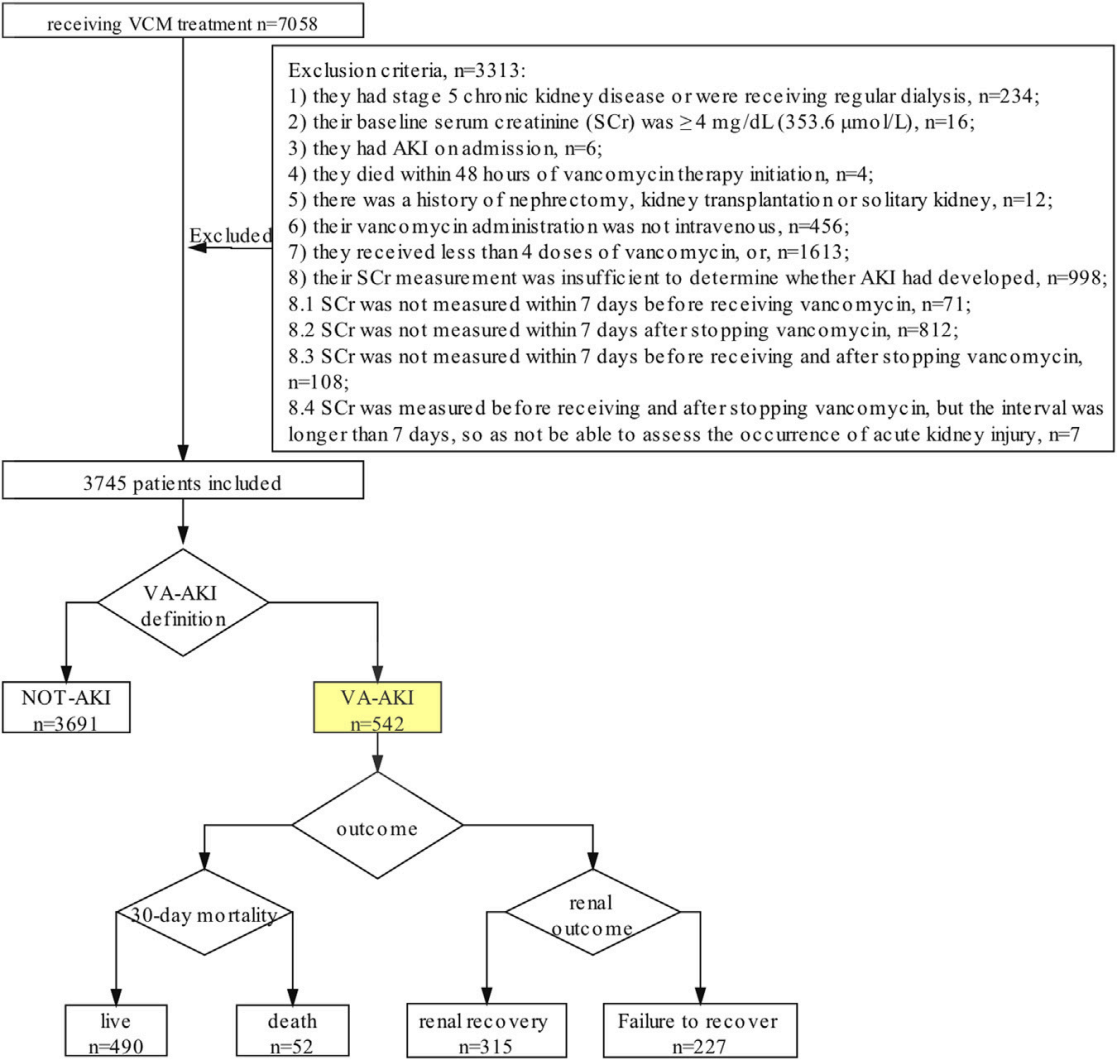
Study Design.

This was a retrospective observational cohort study performed at Zhongshan Hospital Fudan University, a comprehensive, 2005-bed teaching hospital. The survey of VA-AKI was designed to include three steps (Figure 1). First, all adult inpatients treated with vancomycin from January 2016 to June 2019 were evaluated for study inclusion. Patients were excluded if 1) they had stage 5 chronic kidney disease or were receiving regular dialysis; 2) their baseline serum creatinine (SCr) was ≥4 mg/dL (353.6 μmol/L); 3) they had AKI on admission; 4) they died within 48 h of vancomycin therapy initiation; 5) there was a history of nephrectomy, kidney transplantation or solitary kidney; 6) their vancomycin administration was not intravenous; 7) they received less than four doses of vancomycin, or; 8) their SCr measurement was insufficient to determine whether AKI had developed.

Second, we recorded the SCr of the included patients and separated the patients into two groups: the NOT-AKI group and the VA-AKI group. We used the 2012 Kidney Disease:Improving Global Outcomes (KDIGO) definition of AKI as the primary screening criterion, e.g., an increase in SCr by ≥ 0.3 mg/dL (≥26.5 μmol/L) within 48 h or an increase in SCr to ≥1.5 times baseline, which was known or presumed to have occurred within the prior 7 days (KDIGO Acute Kidney Injury Work Group, 2012). VA-AKI was defined as a patient having developed AKI during vancomycin therapy or within 48 h following the withdrawal of vancomycin therapy.

Third, for patients who developed AKI, we further analyzed the severity and outcome of the condition. Severity was assessed based on the highest AKI stage (1, 2, or 3) according to the KDIGO criterion. VA-AKI outcomes for the study included length of hospital stay (LOS), renal recovery, and 30-day mortality rates. Renal recovery was categorized into three levels: full recovery, partial recovery and failure to recover. We defined renal recovery at discharge as full recovery with SCr decreased to the baseline. We defined partial recovery as SCr decreased by 25% or more from peak concentration but remaining higher than baseline. We defined failure to recover as patient still dependent on dialysis or SCr decreased by less than 25% from peak concentration until discharged.

### Data Collection

Data was extracted from the hospital’s electronic database. A researcher uninvolved in the study anonymized patient information. The following variables were collected: demographic information, concomitant underlying diseases, severity of disease, vancomycin exposure, vancomycin variety (Wenkexin vs. Laikexin; trade name: Wenkexin, generic name: Vancomycin Hydrochloride for Injection, manufacturer: VIANEX S.A. (PLANT C), Greece, specification: 500 mg/bottle; and, trade name: Laikexin, generic name: Vancomycin Hydrochloride for Injection, manufacturers: Zhejiang Medicine Co., Ltd. Xinchang Pharmaceutical Factory, China, specification: 500 mg/bottle), therapeutic drug monitoring (TDM) rates, and concomitant nephrotoxic drugs. We also collected data on economic factors and patient outcomes including renal recovery, LOS, and 30- and 90-day mortality rates (Supplementary Table S1).

### Data Analysis

Variables were assessed for normality using the Kolmogorov-Smirnov test. Based on these tests, quantitative variables are presented as means and standard deviations (SDs) or medians and interquartile ranges (IQRs). Variables were then compared between groups using independent t-tests or rank-sum tests. Qualitative variables are presented as frequencies and corresponding percentages and were compared using chi-squared or Fisher’s exact tests.

A multivariate logistic regression analysis was used to assess independent risk factors for VA-AKI occurrence, full renal recovery and mortality. All potential risk factors with a *p* value ≤ 0.05 in the univariate analysis were used in the multiple logistic regression analysis (Supplementary Table S2). A backwards conditional approach was used to enter new terms into the logistic regression. The good of fit was evaluated by the analysis of Hosmer and Lemeshow. All *p* values were two-sided, and a *p* value ≤ 0.05 was considered statistically significant. All statistical analyses were performed using SPSS statistics version 26.0 (IBM Inc., Armonk, NY, United States).

## Results

There were 7058 patients evaluated for study inclusion. After applying the exclusion criteria, 3339 (47.3%) patients were omitted from the study. Of those excluded, 998 patients lacked a SCr measurement, typically within 7 days after receiving vancomycin therapy (Figure 1). A total of 3719 patients were included for analysis. Of these, 66.3% were male and 33.7% were female. The median age was 60 years (IQR, 48.0–68.0).

The incidence of VA-AKI was 14.3% (532/3719) and occurred after 3.0 (IQR, 1.0–7.0) days of treatment. During vancomycin therapy, 86.2% of the patients received at least one nephrotoxic drug. The percentage of patients who received nephrotoxic drugs in combination with vancomycin was 36.8% for one drug, 32.8% for two drugs and 12.5% for three drugs (Supplementary Table S3). The ROC curve analysis indicated that a limit of 1.5 combined nephrotoxic agents was the optimal cut-off value for defining VA-AKI high-risk individuals (Supplementary Figure S1). The most common antibiotic used in combination therapy was carbapenem (58.7%, 2186/3719), while the rate of piperacillin-tazobactam use was 1.6% (62/3719).

### Regional Distribution of the Patients Included

Patients included in the study came from 219 (65.6%, 219/334) municipal boroughs in 30 (88.2%, 30/34) provincial-level administrative regions in China. There are 34 provincial-level administrative regions includes 23 provinces, five autonomous regions, four municipalities and two special administrative regions in China (China Government Network, 2005) (See Supplementary Table S4 for details).

### Therapeutic Drug Monitoring of Patients

According to the vancomycin TDM guidelines issued by the Chinese Pharmacological Society (Ye et al., 2016), 3524 patients were recommended to receive TDM. However, only 1051 (29.8%) patients received it. Monitoring was initiated before the fourth or fifth vancomycin administration in 21.5% patients. A steady state valley concentration between 10 and 20 μmol/L occurred in 42.6% patients, while 27.6% had a concentration greater than 20 μmol/L. The highest monitoring rates occurred in patients with hepatic insufficiency (48.2%) and renal insufficiency (45.5%) (Supplementary Table S5).

### Comparison of Risk Factors Between Patients with and Without VA-AKI

Table 1 displays patient demographic information, concomitant underlying diseases, and severity of illness. Table 2 lists patient vancomycin exposure and concomitant nephrotoxic drugs. The multivariable logistic regression of factors for development of VA-AKI can be seen in Table 3. Patients with VA-AKI were more likely to concomitant with BMI ≥ 30 kg/m^2^ (OR 1.64, 95% CI 1.00–2.69, *p* = 0.05) than those without VA-AKI. There was no significant difference in age or sex between the two groups.

**TABLE 1:** Demographic information and clinical characteristics of patients with and without VA-AKI.

Demographic information	Patients without VA-AKI N = 3187	Patients with VA-AKI N = 532	*p* value
Gender (male)	2094 (65.7)	372 (69.9)	0.057
Age			0.713
＜60 (years)	1579 (49.6)	259 (48.7)	
≥60 (years)	1608 (50.5)	273 (51.3)	
Body mass index			0.034
＜30 (kg/m^2^)	2617 (82.1)	417 (78.4)	
BMI ≥ 30 (kg/m^2^)	92 (2.9)	24 (4.5)	
Concomitant underlying diseases			
Chronic kidney diseases	34 (1.1)	34 (6.4)	<0.001
Chronic hepatic insufficiency	98 (3.1)	43 (8.1)	<0.001
Hypertension	645 (20.2)	127 (23.9)	0.056
Coronary heart disease	322 (10.1)	60 (11.3)	0.409
Heart failure	29 (0.9)	14 (2.6)	0.001
Atrial fibrillation	302 (9.5)	60 (11.3)	0.194
Valvular heart disease	1160 (36.4)	222 (41.7)	0.018
Chronic obstructive pulmonary disease	52 (1.6)	5 (0.9)	0.229
Diabetes	315 (9.9)	53 (10.0)	0.955
Cancer	767 (24.1)	83 (15.6)	<0.001
Anaemia	111 (3.5)	14 (2.6)	0.313
Severity of illness			
Admission to the ICU	1194 (37.5)	282 (53.0)	<0.001
Shock or concomitant vasopressors	345 (10.8)	149 (28.0)	<0.001
Trauma	7 (0.2)	1 (0.2)	1.000^a^
Cardiac surgery	1339 (42.0)	276 (51.9)	<0.001
Major non-cardiac surgery	279 (8.8)	49 (9.2)	0.731
Sepsis	1135 (35.6)	208 (39.1)	0.121

^a^ The calibration of the chi-square test. F refers to Fisher’s exact test.

Data are described as mean (SD), n (%), or median (IQR); VA-AKI, vancomycin-associated kidney injury; ICU, intensive care unit.

**TABLE 2:** Vancomycin exposure and concomitant nephrotoxic drugs of patients with and without VA-AKI.

	Patients without VA-AKI N = 3187	Patients with VA-AKI N = 532	*p* value
Vancomycin exposure			
Vancomycin varieties			<0.001
Wen Kexin	1937 (60.8)	409 (76.9)	
Lai Kexin	1250 (39.2)	123 (23.1)	
Length of vancomycin therapy			<0.001
<7 days	1732 (54.3)	224 (42.1)	
≥7 days and <14 days	1038 (32.6)	182 (34.2)	
≥14 days	417 (13.1)	126 (23.7)	
Dose			0.055^a^
<4 g/d	3186 (100.0)	530 (99.6)	
≥4 g/d	1 (0.03)	2 (0.4)	
Concomitant nephrotoxic drugs			
Aminoglycoside antibiotics	19 (0.6)	2 (0.4)	0.530
Antiviral drugs	103 (3.2)	22 (4.1)	0.284
Rifampin	39 (1.2)	14 (2.6)	0.011
Quinolone antibiotics	62 (2.0)	11 (2.1)	0.851
Sulfonamides	42 (1.3)	11 (2.1)	0.177
β- Lactam antibiotics			<0.001
Vancomycin monotherapy	1001 (31.4)	108 (20.3)	
Cephalosporin	462 (14.5)	98 (18.4)	
Carbapenems	1678 (52.7)	310 (58.3)	
Piperacillin-tazobactam	46 (1.4)	16 (3.0)	
Loop diuretic	1400 (43.9)	294 (55.3)	<0.001
Cyclosporine A	15 (0.5)	3 (0.6)	1.000^b^
Tacrolimus	35 (1.1)	34 (6.4)	<0.001
Chemotherapy	9 (0.3)	1 (0.2)	1.000^b^
Radiocontrast agents	70 (2.2)	29 (5.5)	<0.001
Reninangiotensin system blockers	495 (15.5)	37 (7.0)	0.443
NSAIDs	121 (3.8)	17 (3.2)	0.497

^a^ Fisher’s exact test.

^b^ The calibration of the chi-square test.

Data are described as mean (SD), n (%), or median (IQR). NSAIDs = Non-steroidal anti-inflammatory drugs. The number of concomitant amphotericin B or traditional Chinese medicine was zero. ICU, intensive care unit; VA-AKI, vancomycin-associated kidney injury.

**TABLE 3:** Multivariable logistic regression of factors for development of VA-AKI.

	B	S.E.	OR	95% CI. for OR		*p* value
				Lower	Upper	
Body mass index (≥30 kg/m^2^)	0.50	0.25	1.64	1.00	2.69	0.05
Chronic kidney diseases	1.70	0.34	5.49	2.82	10.68	< 0.001
Chronic hepatic insufficiency	0.89	0.26	2.42	1.45	4.04	0.001
Admission to the ICU	0.37	0.11	1.44	1.15	1.80	0.001
Circulatory shock or vasopressors	0.85	0.13	2.35	1.80	3.05	< 0.001
Cardiac surgery	0.36	0.13	1.43	1.11	1.84	0.005
Vancomycin varieties (Wen Kexin)	0.56	0.13	1.76	1.37	2.25	< 0.001
LOT < 7 days						<0.001
LOT ≥ 7 days and ＜14 days	0.26	0.13	1.30	1.01	1.67	0.043
LOT ≥14 days	0.79	0.15	2.20	1.64	2.94	< 0.001
β-Lactam antibiotics (none)						0.003
β-Lactam antibiotics (Cephalosporin)	0.44	0.18	1.55	1.08	2.21	0.017
β-Lactam antibiotics (Carbapenems)	0.38	0.14	1.46	1.11	1.91	0.006
β-Lactam antibiotics (PTZ)	1.14	0.37	3.12	1.50	6.49	0.002
Tacrolimus	1.07	0.30	2.92	1.63	5.22	< 0.001
Radio-contrast agents	0.92	0.25	2.51	1.55	4.07	< 0.001
Constant	−2.91	0.16	0.06			< 0.001

LOT, Length of vancomycin therapy. ICU, intensive care unit. PTZ, Piperacillin and tazobactam. VA-AKI, vancomycin-associated kidney injury.

More patients in VA-AKI group had concomitant chronic kidney disease (OR 5.49, 95% CI 2.82–10.68, *p* < 0.001) or chronic hepatic insufficiency (OR 2.42, 95% CI 1.45–4.04, *p* = 0.001) and were more likely to have concomitant heart failure (2.6 vs. 0.9%, *p* = 0.001) and valvular heart disease (41.7% vs. 36.4%, *p* = 0.018), but less likely to have cancer (15.6 vs. 24.1%, *p* < 0.001). Patients in VA-AKI group were also more likely to be admitted to the ICU (OR 1.44, 95% CI 1.15–1.80, *p* = 0.001), to experience shock or be given concomitant vasopressors (OR 2.35, 95% CI 1.80–3.05, *p* < 0.001) and undergo cardiac surgery (OR 1.43, 95% CI 1.11–1.84, *p* = 0.005).

Patients with VA-AKI received more Wen Kexin (vs. Lai Kexin) (OR 1.76, 95% CI 1.37–2.25, *p* < 0.001), compared with those without VA-AKI. In addition, patients in the VA-AKI group underwent a longer therapy course. Exposure to loop diuretics (5.5 vs. 2.2%, *p* < 0.001), tacrolimus (OR 2.92, 95% CI 1.63–5.22, *p* < 0.001), and radio-contrast agents (OR 2.51, 95% CI 1.55–4.07, *p* < 0.001) were also more frequent in the VA-AKI group. Furthermore, patients with VA-AKI were more likely to concomitant with concomitant administration of cephalosporin (OR 1.55, 95% CI 1.08–2.21, *p* = 0.017), carbapenems (OR 1.46, 95% CI 1.11–1.91, *p* = 0.006) and piperacillin-tazobactam (OR 3.12, 95% CI 1.50–6.49, *p* = 0.002).

### Comparison of Medical Costs and Outcomes for Patients with and Without VA-AKI

Patients with VA-AKI were more likely to have higher medication costs (6.1 vs. 3.6 thousand US dollars, *p* < 0.001), treatment costs (0.7 vs. 0.4 thousand US dollars, *p* < 0.001) and total costs (19.2 vs. 12.7 thousand US dollars, *p* < 0.001). Patients in the VA-AKI group also had longer hospital stays (23 vs. 20 days, *p* < 0.001) and a higher 30-days mortality rate (8.8% vs. 1.5%, *p* < 0.001) (Table 4).

**TABLE 4:** Medical costs and outcomes of patients with and without VA-AKI.

	Patients without	Patients with	*p* value
	VA-AKI N = 3187	VA-AKI N = 532	
Treatment costs (thousand US$)	0.4 (0.04)	0.7 (0.1)	<0.001
Consumables costs (thousand US$)	4.3 (0.9)	7.5 (1.1)	<0.001
Total costs (thousand US$)	12.7 (1.3)	19.2 (1.9)	<0.001
Length of hospital stay (day)	2.9 (0.2)	3.3 (0.3)	<0.001
30-day mortality	49 (1.5)	47 (8.8)	<0.001
90-day mortality	71 (2.2)	56 (10.5)	<0.001

Data are described as mean (SD), n (%), or median (IQR). b refers to the calibration of the chi-square test. F refers to Fisher’s exact test; VA-AKI, vancomycin-associated kidney injury.

### Severity and Outcomes of VA-AKI Patients

There were 343 VA-AKI patients (64.5%) with KDIGO stage 1 AKI. Thirty-eight patients (7.1%) received dialysis, and those with stage 3 VA-AKI experienced the highest dialysis rate (29.2%).

The 30-day mortality rate of the VA-AKI patients was 8.8%, and 29.8% (14/47) of patients had SCr within the normal range (44–115 μmol L^−1^) at the time of death. For patients with stage 3 AKI the mortality was 16.9%.

58.6% (312/542) of VA-AKI patients have a renal recovery (full recovery or partial recovery), of which 40.2% (218/542) patients fully recovered. The median time to renal recovery is 4.1 (IQR = 5.0) days after VA-AKI occur. Patients with stage 1 AKI had the highest renal recovery rate (46.9%) (Table 5).

**TABLE 5:** Outcomes of VA-AKI patients.

Patient outcomes	Total	Stage 1	Stage 2	Stage 3	*p* value
	N = 532	N = 343	N = 100	N = 89	
30-day mortality n (%)	47 (8.8)	25 (7.3)	7 (7.0)	15 (16.9)	0.014
Receive dialysis n (%)	38 (7.1)	8 (2.3)	4 (4.0)	26 (29.2)	<0.001
Renal recovery n (%)	312 (58.6)	211 (61.5)	56 (56.0)	45 (50.6)	<0.001
Full recovery n (%)	218 (41.0)	161 (46.9)	30 (30.0)	27 (30.3)	0.001
Partial recovery n (%)	94 (17.7)	50 (14.6)	26 (26.0)	18 (20.2)	<0.001
Failure to recover n (%)	220 (41.4)	132 (38.5)	44 (44.0)	44 (49.4)	<0.001

### Risk Factors for Mortality of VA-AKI Patients

Multiple logistic regression analysis revealed that gender (male) (OR 3.053, *p* = 0.035) and age (≥60 years) (OR 3.13, *p* = 0.007) were independent risk factors for mortality. Compared with AKI stage 1, AKI stage 3 (OR 3.352, *p* = 0.007) was an independent risk factor for mortality. Full renal recovery (OR 0.208, *p* = 0.005) and admission to the ICU (OR 0.414, *p* = 0.034) were independent protective factors for mortality (Table 6).

**TABLE 6:** Multivariable logistic regression of factors for mortality of VA-AKI patients.

	B	S.E.	OR	95% CI. for OR		*p* value
				Lower	Upper	
Gender (male)	1.116	0.528	3.053	1.084	8.597	0.035
Age ≥60 (years) vs. <60 (years))	1.141	0.425	3.130	1.361	7.198	0.007
Admission to the ICU	−0.881	0.416	0.414	0.183	0.936	0.034
Cardiac surgery	−0.814	0.427	0.443	0.192	1.023	0.057
AKI Stage 1 (reference)						0.020
AKI Stage 2	0.079	0.562	1.082	0.359	3.258	0.888
AKI Stage 3	1.21	0.448	3.352	1.392	8.071	0.007
Full renal recovery (vs. fail to full renal recover)	−1.572	0.564	0.208	0.069	0.627	0.005
Constant	−3.115	0.652	0.044			0.000

ICU, intensive care unit; VA-AKI, Vancomycin-associated kidney injury.

### Risk Factors for Fail to Full Renal Recovery of VA-AKI Patients

Multiple logistic regression analysis revealed that cancer (OR 2.447, *p* = 0.004) was an independent risk factor for fail to full renal recovery. Compared with AKI stage 1, AKI stage 2 (OR 2.174, *p* = 0.005) and AKI stage 3(OR 2.210, *p* = 0.005) were independent risk factors for fail to full renal recovery. Admission to the ICU (OR 0.626, *p* = 0.023) and shock or concomitant vasopressors (OR 0.526, *p* = 0.003) were independent protective factors for fail to full renal recovery (Table 7).

**TABLE 7:** Multivariable Logistic Regression of Factors for full renal recovery of VA-AKI patients.

	B	S.E.	OR	95% CI. For OR		*p* value
				Lower	Upper	
Cancer	0.895	0.311	2.447	1.331	4.499	0.004
Admission to the ICU	−0.469	0.206	0.626	0.417	0.938	0.023
Shock or concomitant vasopressors	−0.643	0.219	0.526	0.342	0.807	0.003
AKI Stage 1 (reference)						0.001
AKI Stage 2	0.777	0.278	2.174	1.260	3.749	0.005
AKI Stage 3	0.793	0.283	2.210	1.270	3.846	0.005
Constant	0.294	0.188	1.342			0.117

ICU, intensive care unit; VA-AKI, vancomycin-associated kidney injury.

## Discussion

This single-center cohort study, including 3719 patients from 30 of 34 provincial-level administrative regions in China, have investigated the burden and characteristics of VA-AKI in China. Our survey, with to our knowledge, the largest sample size so far and covering patients from different areas in China, further uncovered the risk factors for prognosis of VA-AKI patients.

Our results showed that the incidence of VA-AKI was 14.3%, however, this could be an underestimate as 998 patients were excluded due to insufficient SCr measurements, which is consistent with our previous research (Pan et al., 2018). Therefore, our results may have missed a number of VA-AKI patients.

Currently, TDM is an effective measure used to reduce VA-AKI. However, we found several issues with its use, including an insufficient monitoring rate of the target population, inappropriate TDM start times, and an insufficient rate of achieving steady-state concentrations. The TDM guidelines for vancomycin was issued by the American Society of Health-System Pharmacists in 2009 (Rybak et al., 2009), updated in 2020 (Rybak et al., 2020), and issued by the Chinese Pharmacological Society in 2016 (Ye et al., 2016). However, a survey of vancomycin TDM involving 214 medical institutions in China revealed that vancomycin-monitoring technology, while adequately advanced, was not standardized for monitoring time or target populations in clinical practice (Zhou et al., 2019). This may be due to clinicians in China having high-work loads leading to time constraints and distractions (Jiang et al., 2019).

A complete diagnosis of AKI by SCr measurements and standardized vancomycin TDM is necessary for its management. The most current 2020 guidelines recommend using Bayesian-derived AUC monitoring rather than trough concentrations (Rybak et al., 2020). Several studies have shown that pharmacists who lead or participate in vancomycin medication management programs are conducive in improving the effective use of vancomycin and reducing the mean duration of vancomycin therapy and medical expenses (Willis and Jhaveri, 2018; Dadzie et al., 2019; Porter et al., 2019). Therefore, clinical pharmacists may be able to reduce both the workload of doctors and medical expenses (Willis and Jhaveri, 2018). Thus, hospital administrators should consider increasing their investment in clinical pharmacists to reduce the incidence of VA-AKI.

One distinct feature of this study was the high proportion of concomitant nephrotoxic medication use (86.2%) compared with the 28–71% reported in hospitals from the United States (Hidayat et al., 2006; Choi et al., 2017). Previous investigations have indicated that a combination of vancomycin and nephrotoxic agents is associated with nephrotoxicity (Castanheira et al., 2016; Yang, 2017). Uekl et al. showed that the number of combined nephrotoxic agents (OR, 1.590, *p* = 0.010) was significantly related to nephrotoxicity (Ueki et al., 2020). In accordance with these results, we also observed a significantly higher incidence of VA-AKI in patients given combined multiple nephrotoxic drugs, especially combinations of more than two drugs.

Another distinct finding of this Chinese VA-AKI study was the high proportion of the combined use of carbapenems (especially mipenem and meropenem) with vancomycin, rather than piperacillin-tazobactam (59.3 vs. 1.7%). Vancomycin and piperacillin-tazobactam are among the most commonly prescribed antibiotics in hospitals in the United States, and this particular combination of antibiotics may be empirically useful due to the broad Gram-positive activity of vancomycin and broad Gram-negative activity of piperacillin-tazobactam (Carreno et al., 2018). Both piperacillin-tazobactam and carbapenems have broad Gram-negative activity and are recommended in clinical practice guidelines in China (Ran et al., 2019; Wu and Ren, 2020). We speculate that one reason for more combinations with carbapenems is that piperacillin-tazobactam requires a skin test before administration in China, while carbapenem antibiotics do not. The concern is that penicillin-based antibiotics may cause severe allergic reactions such as anaphylactic shock (Yang, 2017). Therefore, the People's Republic of China Pharmacopoeia Clinical Medication Instructions require skin tests before using penicillin (Yang, 2017). Hence, carbapenem antibiotics are preferred as they are more convenient. Another possible reason for more combinations with carbapenems is the higher prevalence of extended-spectrum beta-lactamase (ESBL) in China, compared to United States. One study that collected 15,588 *Enterobacteriaceae* isolates from 63 hospitals in the United States from 2012 to 2014, found a prevalence of ESBL-producing strains of 13.6% for *Escherichia coli*, 17.4% for *Klebsiella pneumoniae*, 10.8% for *Klebsiella. oxytoca* and 5.7% for *Proteus mirabilis* (5.7%) (Castanheira et al., 2016). In contrast, in 2014 the China Antimicrobial Surveillance Network collected 78,955 Enterobacteriaceae isolates from 15 general hospitals and two children’s hospitals and found that the prevalence of ESBL-producing strains was 55.3% for *E. coli*, 22.9% for *K. pneumoniae* and *K. oxytoca*, and 24.7% for *P. mirabilis* (Hu et al., 2016). Carbapenem antibiotics produce strong antibacterial activity against ESBL-producing strains and are currently the most effective and reliable antibacterial drugs for the treatment of various infections caused by ESBL-producing Enterobacteriaceae bacteria (Zhou et al., 2014).

The combination of vancomycin plus piperacillin-tazobactam increases the odds of inducing AKI, thus vancomycin plus carbapenems may contribute to a lower rate of VA-AKI in China (Balci et al., 2018; Carreno et al., 2018; Ide et al., 2019; Avedissian et al., 2020; Ciarambino et al., 2020). Our multiple regression analysis showed that both carbapenem and piperacillin-tazobactam antibiotics were independent risk factors for VA-AKI, and the OR value of piperacillin-tazobactam was higher than carbapenems (OR = 3.12 vs. OR = 1.46), which is consistent with previous reports (Ide et al., 2019). The potential mechanism underlying the enhanced toxicity of this combination remains uncertain (Gomes et al., 2014). has suggested that subclinical interstitial nephritis caused by piperacillin-tazobactam in combination with the oxidative stress of vancomycin might lead to increased renal injury (Gomes et al., 2014). (Burgess and Drew, 2014) has posited that piperacillin-tazobactam might reduce vancomycin clearance, resulting in increased exposure in the kidney and, hence, further injury (Burgess and Drew, 2014). Therefore, from the perspective of reducing VA-AKI, the combined use of carbapenem antibiotics, rather than piperacillin-tazobactam should be considered a better choice.

Our study had several strengths. First, the sample size was relatively large, and the population was geographically widely distributed. Second, in terms of nephrotoxic drugs, we included categories that were as comprehensive as possible. Third, we gathered data regarding associated medical costs, which has rarely been addressed in the literature. This study also had some limitations. Due to the retrospective design, we were only able to show associations between vancomycin and AKI and not causality. In addition, urine output was not assessed and this may have affected the rates of identified AKI. Furthermore, as trough levels were not drawn for every patient, we were unable to evaluate the potential effect of vancomycin concentration on the development of AKI, which is a well-known risk factor for nephrotoxicity.

VA-AKI is associated with a higher medical expenses and risk of mortality. We carried out this longitudinal study to further analyze the factors that affect the prognosis of patients with VA-AKI, which was rarely involved in previous studies. Our research shows that full renal recovery is an independent protective factor for mortality. Approximately 70% of patients died with impaired renal function, and we speculate that the deaths of these patients may be related to AKI. Compared with unchangeable factors such as gender and age, renal recovery is a risk factor that can be improved, so it is the focus of efforts to reduce the mortality of patients. Only 41.2% of the patients with VA-AKI recovered renal function during hospitalization, which is lower than the 58–81% reported in developed countries (Pritchard et al., 2010; Lacave et al., 2017). Once the patient develops AKI, we recommend prompt and active treatment. Admission to ICU helps improve the patient's full renal recovery reduce mortality. We speculate that patients in the ICU can receive more comprehensive monitoring and timely treatment. Higher AKI stages are independent risk factors of failure to full renal recovery and mortality, which is consistent with previous studies (Forni et al., 2017). In conclusion, it may be necessary to suspend vancomycin or adjust the dosage in a timely manner for the renal recovery (Rybak et al., 2020), especially for patients with high KDIGO AKI stages.

## Conclusion

Lack of a serum creatinine measurement for the diagnosis of AKI and lack of standardization of vancomycin therapeutic drug monitoring should be improved. Patient concomitant with piperacillin-tazobactam are at higher risk. Full renal recovery was associated with a significantly reduced morality.

## Data Availability

The raw data supporting the conclusions of this article will be made available by the authors, without undue reservation. Requests to access the datasets should be directed to the corresponding Author.
