# Supplementary material for: Vancomycin Associated Acute Kidney Injury: A Longitudinal Study in China
Source: Front Pharmacol. 2021 Mar 8;12:632107. doi: 10.3389/fphar.2021.632107 (PMC7982802; doi:10.3389/fphar.2021.632107)
Supplement: Supplementary file 3 [file table1.docx]

Supplementary Table 1. Demographic, economic and clinical variables collected for the study

| **Demographic information** | Gender (male vs. female) |
| --- | --- |
|  | Age (years old) |
|  | Body Mass Index (Kg/m^2^) |
| **Concomitant underlying diseases** | Chronic kidney diseases (yes vs. no) |
|  | Chronic hepatic insufficiency (yes vs. no) |
|  | Hypertension (yes vs. no) |
|  | Coronary heart disease (yes vs. no) |
|  | Heart failure (yes vs. no) |
|  | Atrial fibrillation (yes vs. no) |
|  | Valvular heart disease (yes vs. no) |
|  | Chronic obstructive pulmonary disease (yes vs. no) |
|  | Diabetes (yes vs. no) |
|  | Cancer (yes vs. no) |
|  | Anemia (yes vs. no) |
| **Severity of illness** | Admission to the ICU (yes vs. no) |
|  | Shock or concomitant vasopressors (yes vs. no) |
|  | Trauma (yes vs. no) |
|  | Cardiac surgery (yes vs. no) |
|  | Major noncardiac surgery (yes vs. no) |
|  | Sepsis (yes vs. no) |
| **Vancomycin exposure** | Vancomycin varieties (Wen Kexin vs.Lai Kexin) |
|  | Length of therapy (yes vs. no) |
|  | Dose (mg) |
| **Nephrotoxic drugs** | No.1 Aminoglycoside antibiotics: gentamicin, kanamycin, amikacin, tobramycin, streptomycin, neomycin) |
|  | No.2 amphotericin B |
|  | No.3 Antiviral drugs: adefovir dipivoxil, cidofovir, tenofovir, foscarnet, acyclovir, ganciclovir, valganciclovir, valacyclovir, indinavir, interferon, atazanavir |
|  | No.4 Calcineurin inhibitor: cyclosporine A, tacrolimus |
|  | No.5 Chemotherapy: cisplatin, carboplatin, gemcitabine, methotrexate, cyclophosphamide |
|  | No.6 Radiocontrast agents |
|  | No.7 Vasopressors: epinephrine, milrinone, dopamine, vasopressin |
|  | No.8 loop diuretics: Furosemide, torasemide |
|  | No.9 reninangiotensin system blockers: Angiotensin-Converting Enzyme Inhibitors, Angiotensin Receptor Blockers |
|  | No.10 β-Lactam antibiotics：Cephalosporins, carbapenems, piperacillin + tazobactam |
|  | No.11 Non-steroidal anti-inflammatory drugs |
|  | No.12 rifampin |
|  | No.13 ciprofloxacin |
|  | No.14 sulfonamides |
|  | No.15 Traditional Chinese medicine (cantharidin, venenum bufonis, realgar, radix aconiti kusnezoffi,raw rhizoma typhonii, caulis aristolochiae manshuriensis, aristolochia fangchi, radix aristolochiae, fibraurea recisa pierre, herba aristolochiae mollissimae, aristolochia, Ciliatenerve knotweed root and other nephrotoxic traditional Chinese medicine) |
| **Economic factors** | Treatment costs (thousand US$) |
|  | Consumables costs (thousand US$) |
|  | Total costs (thousand US$) |
| **Patient outcomes** | Renal recovery (yes vs. no) |
|  | Length of hospital stay (yes vs. no) |
|  | 30-day mortality (yes vs. no) |
|  | 90-day mortality (yes vs. no) |
