# Supplementary material for: Vancomycin Associated Acute Kidney Injury: A Longitudinal Study in China
Source: Front Pharmacol. 2021 Mar 8;12:632107. doi: 10.3389/fphar.2021.632107 (PMC7982802; doi:10.3389/fphar.2021.632107)
Supplement: Supplementary file 4 [file table2.docx]

Supplementary Table 2. Factors used in the multiple logistic regression

| **For development of VA-AKI** | Gender (male)  Age (＜60 years old vs. ≥60 years old)  Body Mass Index (＜30 Kg/m^2^ vs. ≥30 Kg/m^2^ )  Chronic kidney diseases (yes vs. no)  Chronic hepatic insufficiency (yes vs. no)  Heart failure (yes vs. no)  Valvular heart disease (yes vs. no)  Cancer (yes vs. no)  Admission to the ICU (yes vs. no)  Cardiac surgery (yes vs. no)  Vancomycin varieties (Wen Kexin vs.Lai Kexin)  Length of vancomycin therapy (＜7 days vs. ≥7 days & ＜14 days, ≥14 days)  Rifampin (yes vs. no)  β - Lactam antibiotics (Vancomycin monotherapy vs. Cephalosporin, Carbapenems, Piperacillin-tazobactam)  Loop diuretic (yes vs. no)  Tacrolimus (yes vs. no)  Radiocontrast agents (yes vs. no) |
| --- | --- |
| **For morality** | Gender (male)  Age (＜60 years old vs. ≥60 years old)  Body Mass Index (＜30 Kg/m^2^ vs. ≥30 Kg/m^2^)  Valvular heart disease (yes vs. no)  Admission to the ICU (yes vs. no)  Cardiac surgery (yes vs. no)  Length of vancomycin therapy (＜7 days vs. ≥7 days & ＜14 days, ≥14 days)  Loop diuretic (yes vs. no)  Acute kidney injury stage (stage 1, stage 2, stage3)  full renal recovery(vs. Fail to full renal recovery) |
| **For full renal recovery** | Gender (male)  Age (＜60 years old vs. ≥60 years old)  Body Mass Index (＜30 Kg/m^2^ vs. ≥30 Kg/m^2^)  Valvular heart disease (yes vs. no)  Cancer (yes vs. no)  Admission to the intensive care unit (yes vs. no)  Shock or concomitant vasopressors (yes vs. no)  Cardiac surgery (yes vs. no)  Tacrolimus (yes vs. no)  Acute kidney injury stage (stage 1, stage 2, stage3) |

VA-AKI: Vancomycin associated acute kidney injury
