# Supplementary material for: Vancomycin Associated Acute Kidney Injury: A Longitudinal Study in China
Source: Front Pharmacol. 2021 Mar 8;12:632107. doi: 10.3389/fphar.2021.632107 (PMC7982802; doi:10.3389/fphar.2021.632107)
Supplement: Supplementary file 5 [file table3.docx]

Supplementary Table 3 Number of concomitant nephrotoxic drugs

| **Number of concomitant nephrotoxic drugs** | **Number of patients** | **Percentage of patients** | **Number of patients developed VA-AKI** | **Incidence of VA-AKI** |
| --- | --- | --- | --- | --- |
| 0 | 512 | 13.8% | 50 | 9.8% |
| 1 | 1369 | 36.8% | 122 | 8.9% |
| 2 | 1218 | 32.8% | 187 | 15.4% |
| 3 | 464 | 12.5% | 113 | 24.4% |
| 4 | 123 | 3.3% | 43 | 35.0% |
| 5 | 29 | 0.8% | 16 | 55.2% |
| 6 | 4 | 0.1% | 1 | 25.0% |

VA-AKI: Vancomycin associated acute kidney injury
