# Supplementary material for: Vancomycin Associated Acute Kidney Injury: A Longitudinal Study in China
Source: Front Pharmacol. 2021 Mar 8;12:632107. doi: 10.3389/fphar.2021.632107 (PMC7982802; doi:10.3389/fphar.2021.632107)
Supplement: Supplementary file 6 [file table4.docx]

Supplementary table 4 Distribution of the provincial districts.

| **Provincial districts Name** | **N** | **%** |
| --- | --- | --- |
| ShangHai City | 1223 | 33.3% |
| JiangSu Province | 860 | 23.4% |
| ZheJiang Province | 523 | 14.2% |
| AnHui Province | 375 | 10.2% |
| JiangXi Province | 170 | 4.6% |
| FuJian Province | 89 | 2.4% |
| HeNan Province | 62 | 1.7% |
| ShanDong Province | 59 | 1.6% |
| HuBei Province | 38 | 1.0% |
| HeiLongjiang Province | 31 | 0.8% |
| SiChuan Province | 31 | 0.8% |
| HuNan Province | 28 | 0.8% |
| GanSu province | 25 | 0.7% |
| GuiZhou Province | 22 | 0.6% |
| XinJiang Uygur Autonomous Region | 21 | 0.6% |
| YunNan Province | 15 | 0.4% |
| ChongQing City | 14 | 0.4% |
| LiaoNing Province | 14 | 0.4% |
| JiLin Province | 14 | 0.4% |
| GuangDong Province | 10 | 0.3% |
| ShanXi Province | 10 | 0.3% |
| Inner Mongolia Autonomous Region | 7 | 0.2% |
| GuangXi Zhuang Autonomous Region | 6 | 0.2% |
| Ningxia Hui Autonomous Region | 6 | 0.2% |
| ShanXi Province | 5 | 0.1% |
| HeBei Province | 5 | 0.1% |
| China Hong Kong | 4 | 0.1% |
| BeiJing City | 3 | 0.1% |
| TianJin City | 2 | 0.1% |
| QingHai Province | 2 | 0.1% |
| Total | 3674 | 100% |
