# Supplementary material for: Vancomycin Associated Acute Kidney Injury: A Longitudinal Study in China
Source: Front Pharmacol. 2021 Mar 8;12:632107. doi: 10.3389/fphar.2021.632107 (PMC7982802; doi:10.3389/fphar.2021.632107)
Supplement: Supplementary file 7 [file table5.docx]

Supplementary Table 5 The therapeutic drug monitoring (TDM) situation of patients included

| **Patients who are recommend to receive TDM** | **Total (n)** | **Receive TDM (n)** | **%** |
| --- | --- | --- | --- |
| Patients who receive concomitant nephrotoxic agents | 3207 | 986 | 30.7% |
| Intensive care unit admissions | 1476 | 571 | 38.6% |
| Body mass index＞30 Kg/m^2^ | 116 | 38 | 32.7% |
| Those who have burns or impaired renal function | 68 | 31 | 45.5% |
| Elderly patients (≥60 years old) | 1311 | 570 | 30.3% |
| Patients with concomitant hepatic diseases | 141 | 68 | 48.2% |
| Total | 3583 | 1067 | 29.7% |

Patients' TDM recommendations are according to the TDM Guidelines of VCM issued by the Chinese Pharmacological Society.
