# Supplementary material for: Vancomycin Associated Acute Kidney Injury: A Longitudinal Study in China
Source: Front Pharmacol. 2021 Mar 8;12:632107. doi: 10.3389/fphar.2021.632107 (PMC7982802; doi:10.3389/fphar.2021.632107)
Supplement: Supplementary file 8 [file table6.docx]

Supplementary Table 6 Outcomes of VA-AKI patients

| **Patient outcomes** | **Total**  **N = 532** | **Stage 1**  **N = 343** | **Stage 2**  **N = 100** | **Stage 3**  **N = 89** | **P value** |
| --- | --- | --- | --- | --- | --- |
| Receive dialysis n (%) | 38 (7.1) | 8 (2.3) | 4 (4.0) | 26 (29.2) | <0.001 |
| Renal recovery n (%) | 312 (58.6) | 211 (61.5) | 56 (56.0) | 45 (50.6) | <0.001 |
| Full recovery n (%) | 218 (41.0) | 161 (46.9) | 30 (30.0) | 27 (30.3) | 0.001 |
| Partial recovery n (%) | 94 (17.7) | 50 (14.6) | 26 (26.0) | 18 (20.2) | <0.001 |
| Failure to recover n (%) | 220 (41.4) | 132 (38.5) | 44 (44.0) | 44 (49.4) | <0.001 |
